# Intrathoracic splenosis – lesson learned: a case report

**DOI:** 10.1186/s13019-016-0474-3

**Published:** 2016-04-26

**Authors:** Lubomír Tulinský, Peter Ihnát, Marcel Mitták, Petra Guňková, Pavel Zonča

**Affiliations:** Department of Surgery, University Hospital Ostrava, 17.listopadu 1790, Ostrava, 70852 Czech Republic; Department of Surgical Studies, Faculty of Medicine, University of Ostrava, Syllabova 19, Ostrava, 703 00 Czech Republic

**Keywords:** Intrathoracic splenosis, Pleural nodularity, Thoracoscopy, Splenectomy, Case report

## Abstract

**Background:**

Intrathoracic splenosis presents an extremely rare thoracic lesion occurring after a simultaneous rupture of the spleen and diaphragm as a consequence of heterotopic autotransplantation and implantation of splenic tissue. Intrathoracic splenosis is usually an asymptomatic, incidental finding, which should be ideally managed without surgical intervention.

**Case presentation:**

We present a case of 68-year old woman with intrathoracic splenosis. Patient presented with a 2-month history of a dry cough unresponsive to administered antibiotics and antimycotics. Computed tomography (CT) of the chest revealed two homogeneous pleural nodules (diameters of 2 and 4 cm) in the left upper lung field. Two consequent CT-assisted transthoracic core-cut biopsies were performed. Histopathology examination of both biopsy specimens was inconclusive (haemorrhagic and non-specific tissue). After that, patient was referred to the department of thoracic surgery with a suspicion of malignant mesothelioma or metastatic lesions. Thoracoscopic revision of the left pleural cavity was performed and the presence of pleural nodules was confirmed. Bloody looking nodules were resected (standard thoracoscopic resection). Postoperative recovery was uneventful. The histopathology examination of the specimen showed normal splenic tissue. Only with the histopathology report in hand, a detailed medical history was taken. It revealed a gunshot injury requiring splenectomy (without known diaphragm or lung injury) 44 years ago (one of the longest time periods in the literature).

**Conclusions:**

We would like to point out that following the recommendations regarding splenosis may be very difficult in daily routine practice. The simple question regarding abdominal trauma in a patient’s history can lead the clinician to the diagnosis of splenosis, which can be unequivocally established via scintigraphy. The importance of thorough medical history taking, therefore, cannot be underestimated.

## Background

Splenosis presents an infrequent disorder occurring after a splenic trauma as a consequence of heterotopic autotransplantation and implantation of splenic tissue [1, 2]. Splenic implants can be localized anywhere within the peritoneal cavity on the surface of parietal or visceral peritoneum. Intrathoracic localization of splenosis is extremely rare – it is associated with a history of previous simultaneous rupture of the spleen and diaphragm as a result of trauma [2, 3].

Intrathoracic splenosis is usually an incidental finding, asymptomatic; nodular lesion of variable sizes are found in the left pleural cavity [2–4]. The correct diagnosis during preoperative workup is challenging and crucial because it allows for the avoidance of unnecessary surgical resection. On one hand, thoracic nodular lesions of unknown origin should be removed via thoracotomy or video-assisted thoracoscopic surgery according to the guidelines. On the other hand, intrathoracic splenosis removal is not recommended [2–4]. In fact, correct preoperative diagnosis might be difficult, because the condition is rare and thus, not suspected.

Herein, we report a case of a 68-year old woman with intrathoracic splenosis. We would like to point out that following the recommendations regarding splenosis may be very difficult in daily routine practice, and highlight the importance of thorough medical history taking.

## Case presentation

**Fig. 1:**
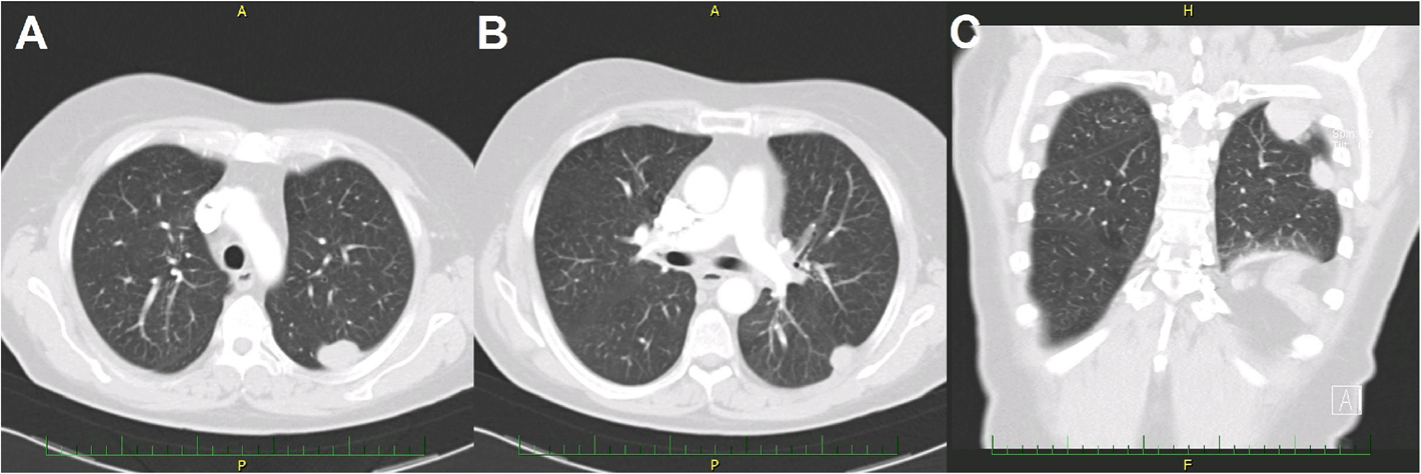
CT scan shows two nodular masses of parietal pleura (**a**, **b** – transversal section, **c** – frontal section)

**Fig. 2:**
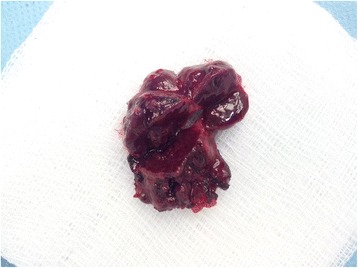
Specimen of splenosis

A 68-year old woman presented with a 2-month history of a dry cough unresponsive to administered antibiotics and antimycotics. According to a referral letter from her general practitioner, she was treated only for rheumatoid arthritis (using methotrexate) for the last 5 years. Computed tomography (CT) of the chest revealed two homogeneous pleural nodules (diameters of 2 and 4 cm) in the left upper lung field (Fig. 1a, b, c). As a result, two consequent CT-assisted transthoracic core-cut biopsies were performed. From both biopsies, the histopathology examination was inconclusive (haemorrhagic and non-specific tissue). After that, patient was referred to our department of thoracic surgery with a suspicion of malignant mesothelioma or metastatic lesions. A minimally invasive revision (thoracoscopy) of the left pleural cavity was performed and the presence of both pleural nodules was confirmed. Bloody looking nodules were resected (standard thoracoscopic resection) and removed via a protective bag. Postoperative recovery was uneventful, the patient was discharged after a few days. The histopathology examination of the specimen showed normal splenic tissue (Fig. 2). Only with the histopathology report in hand, a detailed medical history was taken. It revealed a gunshot injury requiring splenectomy (without known diaphragm or lung injury) 44 years ago. The routine follow-up of the patient showed that the dry cough subsided after several weeks. There were no overwhelming infections or sepsis during the 3 years after the surgery.

### Discussion

The first referral of thoracic splenosis was published by Shaw and Shafi in 1937 [5]. They described a case of splenic tissue autotransplantation into the thoracic cavity after splenectomy because of traumatic injury. In 2003, Yammine reported a total of 37 published case reports of intrathoracic splenosis [6]. During several years, the number of published cases almost doubled – Khan reported 66 cases in 2010 [7]. Currently, approximately 75 cases of intrathoracic splenosis have been referred to in the available English language literature.

According to published data, thoracic splenosis is always located in the left hemithorax, occurring after simultaneous splenic and diaphragmatic injury. The time interval between abdominal injury requiring splenectomy and thoracic splenosis detection varies from several years to decades (trauma is usually remote; the average time being roughly 20 years). In our case, intrathoracic splenosis was found 44 years after splenectomy, which presents one of the longest time periods in the available literature.

The majority of cases of intrathoracic splenosis are asymptomatic; splenosis is usually found incidentally. Only few patients (less than 10–15 % of cases) present with clinical symptoms such as chest pain, hemoptysis or a cough [3, 8–10]. These symptoms (chest pain or cough most frequently) may be caused by mechanical irritation or pressure. This is evidence of the great importance of determination of the proper etiology of clinical symptoms such as chronic cough or thoracic pain.

Our patient suffered from a dry cough for 2 months at the time of referral to our department of thoracic surgery. Antibiotics and antimycotics were administered for 3 weeks, but this treatment was unsuccessful in relieving patient from dry cough. Microscopic evaluation of sputum showed *Burkholderia cepacia* complex. The antibiotics were switched and the patient was treated with third-generation cephalosporin 1 week before the surgery. Preoperative CT scans showed no changes in the lung parenchyma which was suggestive of other lung diseases. Clinical symptoms of our patient completely disappeared after the surgery, therefore postoperative microscopic sputum evaluation was not repeated. Symptom disappearance the surgery would justify this performed thoracoscopic resection. However, the real etiology of dry cough of our patient is questionable – was it mechanical irritation due to intrathoracic splenosis or antibiotic therapy aimed at *Burkholderia cepacia* extermination? Nevertheless, several authors point to the possible role of intrathoracic splenosis as the etiology of dry cough [2, 9, 10].

Besides detailed medical history taking, proper physical examination can help us as well. Left upper quadrant scars should call our attention to possible history of splenic injury. Scars after upper midline laparotomy should be considered suspicious too, because midline laparotomy presents the common way for abdominal revision after gunshot or stab injury. In our case, the initial physical examination revealed only well-healed scar after midline incision; the patient had explained it as an old injury. Only with the histopathology report in hand, a more detailed physical examination revealed a small scar after a gunshot injury in left upper quadrant of the abdomen. Therefore we would like to highlight the importance of proper medical history taking and detailed physical examination.

Asymptomatic thoracic splenosis usually presents as an incidental finding of pleural masses on chest radiograph or CT scans. A solitary nodule (25 % of cases) or multiple nodules are detected on CT scans. CT or MRI images demonstrate exact localization, topographic relationships, and dimensions of the lesions. It is compulsory to differentiate splenosis from a wide spectrum of possible intrathoracic tumors such as primary lung carcinoma, mesothelioma, low-grade lymphoma, thymoma, neurogenic tumors or metastatic lesions [2, 7]. To determine a correct diagnosis of intrathoracic splenosis without surgery, several diagnostic tools are available currently. Haematological markers, such as the absence of Howel-Jolly bodies or siderocytes in peripheral blood, indicate the presence of functional splenic tissue (in patients after splenectomy). If splenosis is suspected, ferumoxide MRI is a helpful tool for diagnosis confirmation. Ferumoxide is a superparamagnetic MRI contrast medium consisting of small molecules of iron oxide which have an affinity for reticuloendothelial tissues – there is a signal loss on T2 sequences of MRI scans [11]. Thoracic splenosis can be confirmed accurately (without biopsy of surgery) by special radionuclide imaging examination. Scintigraphy with Tc-99m-labeled and heat-altered erythrocyte (combined SPECT/CT scanning) is the gold standard for splenic tissue confirmation based on labeled erythrocytes splenic uptake [12]. Radionuclide imaging with Tc-99m sulfur colloid and indium-111-labeled platelets presents an alternative (less sensitive) examination [13]. The correct use of available diagnostic tools can make an accurate diagnosis and avoid risky and unnecessary surgery.

It has been suggested that the implanted splenic tissue offers some level of protection against bacterial infection, lowering the frequency of post-splenectomy sepsis. Outcomes of some studies show a decent improvement of phagocytosis [7, 14, 15]. However, the full immunologic effect of splenosis in asplenic patients remains unknown according to the available literature. Splenosis may not provide sufficient protection against overwhelming infections and sepsis. Connel et al. concluded that splenic nodules usually do not guarantee full immunologic defense, but according to experimental studies, splenosis may generate some protection especially when a large amount of tissue is transplanted [16]. In general, surgical removal of splenosis is not recommended, mainly due to some minimal protection against encapsulated bacterial infection. In an effort to avoid surgical resection, exact diagnosis of nodular lesions of unknown origin is required.

According to National Guidelines Clearinghouse, there are three accepted options in the management of patients with intrathoracic nodules or masses of unknown origin: percutaneous biopsy of pleural masses under CT guidance, fluorine-18-fluoro-2-deoxy-D-glucose - positron emission tomography (FDG-PET) whole body scan, and surgical resection [17]. Imaging only follow-up and conservative management are not recommended. Percutaneous biopsy under CT guidance is currently considered to be the first line procedure. However, CT guided biopsies may be inconclusive or may even lead to the wrong diagnosis [18, 19]. Hemorrhage complicates approximately 1 % of biopsies [20], but in case of well-perfused tissue (such as splenic tissue), the risk of bleeding increases significantly and may outweigh the benefit of percutaneous biopsy. Surgical resection offers some benefits – the guarantee of an adequate and definitive histopathology evaluation and outcome being the most important one.

In our institution, patients with intrathoracic nodules or masses of unknown origin are indicated for percutaneous biopsy under CT. After a failed (inconclusive) biopsy, surgical resection is performed by means of videothoracoscopy, VATS (video- assisted thoracoscopic surgery), or thoracotomy. The selection of surgical approach depends on the lesion size and localization. Minimally invasive approaches are preferred; a thoracotomy is reserved for typically large or unapproachable nodules. During the management of our patient, the idea of possible intrathoracic splenosis was highly unexpected. Owing to this, after inconclusive histopathology findings from CT-guided biopsies, surgical resection was performed. If intrathoracic splenosis was suspected, a correct diagnosis would have been confirmed via scintigraphy and unnecessary surgery would have been avoided.

## Conclusions

In conclusion, thoracic splenosis presents an extremely rare condition which should be ideally managed without surgical intervention. In all patients presenting with nodular lesions of unknown origin in the left hemithorax, the possibility of thoracic splenosis should be considered. The simple question regarding abdominal trauma in a patient’s history can lead the clinician to the diagnosis of splenosis, which can be unequivocally established via scintigraphy. The importance of thorough medical history taking, therefore, cannot be underestimated.

## Consent

Written informed consent was obtained from the patient for publication of this report and any accompanying images.
